# Effect of a fiber-rich cabbage outer leaf powder on the physicochemical, rheological, and sensorial characteristics of a set-type yogurt during cold storage

**DOI:** 10.1016/j.fochx.2026.103529

**Published:** 2026-01-13

**Authors:** Suleiman A. Althawab, Hesham Al-Quh, Abdulhakeem Alzahrani, Isam A. Mohamed Ahmed

**Affiliations:** Department of Food Science and Nutrition, College of Food and Agricultural Sciences, King Saud University, P. O. Box 2460, Riyadh 11451, Saudi Arabia

**Keywords:** Bioactive properties, Cabbage outer leaf powder, Dietary fiber, Functional yogurt

## Abstract

This study investigates the impact of cabbage outer leaf powder (COLP) on the quality attributes of a set-type yogurt. COLP is rich in vitamin C (156.62 mg/100 g), total phenolic content (TPC, 217 mg GAE/100 g), minerals, total dietary fiber (TDF, 64.41 g/100 g), and insoluble dietary fiber (IDF, 52.91 g/100 g) and exhibited high DPPH radical scavenging activity (82.81% inhibition). COLP supplementation significantly (*p* < 0.05) increased the ash, total solids, vitamin C, TDF, IDF, soluble dietary fiber (SDF), Fe, K, Mg, Ca, Mn, Na, TPC and DPPH antiradical activity of yogurt. It significantly increased (*p* < 0.05) the acidity, water holding capacity (WHC), hardness, adhesiveness, springiness, cohesiveness, redness (a*), yellowness (b*), color, flavor, taste, and overall acceptability and significantly (*p* < 0.05) decreased the pH, syneresis, and lightness (L*) of yogurt. Overall, COLP improved yogurt's nutritional, bioactive, and physical quality attributes and can be considered a natural additive to produce a functional yogurt without negative effect on sensory attributes.

## Highlights

• Yogurt was fortified with cabbage out layer leaf powder (COLP) to improve its quality.
• COLP is rich of dietary fiber, bioactive compounds, and essential minerals
• COLP improved the physicochemical and bioactive qualities attributes of yogurt.
• COLP is natural additive to develop a functional yogurt with improved quality.

## 1 Introduction

Yogurt is the most popular and widely consumed dairy product throughout the world and its consumption and market demands have greatly expanded in the post-COVID-19 era due to its well proved nutritional and health benefits and probiotic properties (Trajkovska et al., 2025). Consumption of an adequate amount of yogurt can improve minerals bioavailability, immune function, lactose intolerance, and decrease the blood cholesterol and type 2 diabetes risk (Fan et al., 2022; Lordan, 2024). Yogurt is a rich source of minerals, vitamins, prebiotics, probiotics, and bioactive peptides and is thus considered a nutritious and healthy food product, however, it is low in dietary fiber, iron, and phenolic compounds (Ghaderi-Ghahfarokhi et al., 2024; Tian et al., 2024). Ordinary yogurt also has weak gel characteristics and soft structure, which can lead to defects in physical structure and sensory attributes. Therefore, researchers have used several hydrocolloids to enhance the gel structure of yogurt (Zhang et al., 2024). To overcome such shortcomings, and in response to increasing consumer preferences for nutritious and healthy foods, the dairy industry has shifted toward adopting new strategies for producing functional yogurt with enhanced physicochemical and sensory properties (Trajkovska et al., 2025). Fortification of yogurt with natural bioactive compounds and dietary fiber has emerged as a novel approach to overcome the lack of phenolic compounds, dietary fiber, and gel consistency of plain yogurt (Zhang et al., 2024). In this regard, yogurts were fortified with several plant dietary fibers and powders such as carrot soluble dietary fiber (Dong et al., 2022), grape fruit dietary fiber (Qin et al., 2023), kale fiber (Zhang et al., 2024), modified okara insoluble dietary fiber (Tian et al., 2024), pea fiber (Qin et al., 2025), white mulberry powder (Sheikh et al., 2023), white radish powder (Mohamed Ahmed et al., 2024), carrot powder (Hafeez et al., 2025), and hawthorn powder (Wang et al., 2025). These fortified functional yogurts have enhanced nutritional quality, physicochemical properties, sensory attributes, and health benefits and thus it gained increasing popularity and consumer attraction in recent decades (Trajkovska et al., 2025). The dietary fibers of vegetables and fruits wastes are of high potentials importance for developing functional yogurts and studies on the utilization of these valuable wastes in yogurt industry received great attention in recent years.

Vegetables wastes are generated in high quantities worldwide during postharvest processing, trading, handling, and consumption creating high environmental risks as they have high moisture content and nutritionally dense elements, which promote microbial growth and production of hazardous metabolites (Jiménez-Moreno et al., 2020). In Saudi Arabia, large amounts of vegetables (33%) wastes are generated each year putting high pressure on the environment and management systems in addition to economic impacts of these wastes (Alshabanat et al., 2021). Global cabbage production was 73.8 million tons in 2023, whereas Saudi Arabia produced 17,518.2 tons of cabbages annually (FAOSTAT, 2025). Cabbages are rich in nutrients such as bioactive compounds, vitamins, dietary fibers and minerals and have acceptable sensory attributes and great health promotion potentials (Zayed et al., 2023). During postharvest processing, handling, and trading, the cabbages outer leaves (COL) are discarded as wastes in large amounts (39%) creating high environmental concerns despite their great nutritional values (Brito et al., 2021). The COL contain considerable amounts of insoluble dietary fiber, minerals namely potassium and calcium, vitamins, and numerous phenolic compounds and it possess high antioxidant and antimicrobial activities (Brito et al., 2021). Despite great nutritional and therapeutic potentials of COL, still now these valuable byproducts are not fully utilized in yogurt fortification and are on the other hand discarded into the environment leading to high environmental hazards and huge economic and value losses. Therefore, this study was conducted to utilize cabbage outer leaf powder (COLP) as a source of natural polyphenols and dietary fiber and to assess the impact of different concentrations of COLP on the quality characteristics of yogurt stored at 4 °C for 24 days.

## 2 Materials and methods

### 2.1 Materials

Fresh white cabbage (*Brassica oleracea* L. var. *capitata*) was procured from vegetable market in Riyadh city, Saudi Arabia and transported in cooling box to the laboratory. Then, the outer leaves, which commonly discarded as waste, were collected, washed under running tap water, and then lyophilized. The dried leaves were subjected to a milling process (Waring, 8011BU, Torrington, CT) for 1 min at 18,000 rpm and sieved (Retsch, AS200 basic, Haan, Germany) to obtain a fine powder (˂ 450 μm particle size). The powder was vacuum-packed and stored at −20 °C for further use. Milk powder was bought from local market. Lactic acid bacteria starter culture (*Lactobacillus delbrueckii* subsp. *bulgaricus* and *Streptococcus thermophilus*) was procured from Chr. Hansen (YC-X11, Chr. Hansen, Horsholm, Denmark). Sigma-Aldrich (St. Louis, MO, USA) provided all analytical grade chemicals used in this study.

### 2.2 Yogurt making

The concentrations of the cabbage outer leaf powder (COLP) were specified based on preliminary experiments for developing acceptable yogurt in terms of consumer acceptability and physical properties. The designated concentration range (0.5–2.5% COLP) is practically feasible, consistent with ranges frequently reported in the literature, and resulted in acceptable yogurt in preliminary tests. Four treatments (0% COLP, 0.5% COLP, 1.5% COLP, and 2.5% COLP) of a randomized block design was used for yogurt manufacturing process and the whole blocks were replicated (*n* = 3). In this process, 12% milk powder was mixed with different concentrations of COLP followed by adding distilled water, 5 min homogenization (T25 Homogenizer, Ika, Staufen, Germany), and 30 min pasteurization at 85 °C. After that, the mixture was left to cooling down to 42 °C and then inoculation was done by using 2.0% of reactivated starter culture (*L*. *delbrueckii* subsp. *bulgaricus* and *S. thermophilus*). Then, the mixture was aseptically poured into sterilized plastic cups of 14 mL volume and incubated for 4 h (pH 4.6) at the same temperature. After that, yogurt samples were kept at 4 °C and samples were taken periodically at 1, 8, 16, and 24 days of storage for analysis.

### 2.3 Chemical composition analyses

The standard official method (AOAC, 2005) was used for analysis of moisture, total solids, ash, fat, and protein of the samples. Moisture and total solids of yogurt and COLP were assessed by oven drying at 105 °C for overnight (method no. 925.23), ash was measured by ignition at 660 °C in muffle furnace for 6 h (method no. 923.03), protein was assessed by micro-Kjldahl method (method no. 984.13). Fat content of COLP was assessed by Soxhlet method (method no. 920.39) and that of yogurt was measured using Gerber method (method no. 2000.18). Vitamin C contents of COLP and fresh yogurt samples were assessed by 2,6- dichloroindophenol method (AOAC, 2005) after homogenizing 5 g sample in 25 mL metaphosphoric acid (3%, *v*/v) followed by filtration and titration of 5 mL aliquots against 2,6 dichloroindophenol dye and results were expressed as mg ascorbic /100 g FW. The dietary fiber contents of COLP and fresh yogurt samples were measured using an enzymatic–gravimetric procedure method 978.10 (AOAC, 2005) by gelatinizing the samples with α-amylase followed by digestion with amyl glucosidase and protease for removing starch and protein. After that, filtration was done to collect the retentate, which contain the insoluble dietary fiber (IDF), followed by washing with warm distilled water and collection. Then, 4 volumes of 95% ethanol at 60 °C was added to the combined filtrate and washing water for the precipitation of soluble dietary fiber (SDF). The remains were dried at 105 °C until constant weight and then weighed. The residual ash and protein were measured and used for corresponding correction. The sum of SDF and IDF gave the total dietary fiber (TDF). For mineral analysis, to 0.5 g COLP or yogurt samples 5 mL of concentrated sulfuric acid and 3 mL of 30% hydrogen peroxide were added, mixed well, and then subjected to digestion in a Kjeldahl digestion system at 450 °C until all the organic matter was oxidized. After that, the digested samples were cool down and the volume was completed to 100 mL with distilled water in a 100 mL volumetric flask. Minerals were analyzed in the digested samples by using ICP-OES in reference to standard minerals as described previously (Mohamed Ahmed et al., 2020).

### 2.4 Bioactive properties (TPC and DPPH antiradical activity) determination

#### 2.4.1 COLP and yogurt extracts preparation

Water extracts of COLP was prepared by sonication of 2 g COLP in 50 mL distilled water at 110 W constant power, 40 kHz frequency, 60 °C temperature and 30 min sonication time (Mohamed Ahmed et al., 2021). After cooling to ambient temperature, the mixture was subjected to filtration followed by collection of filtrates, re-sonication of the residues in another 50 mL distilled water, and filtration. The filtrates of first and second sonication rounds were recombined. For preparation of yogurt extracts, 100 g yogurt samples were subjected to centrifugation (5000 ×*g*, 4 °C, 5 min) and re-centrifugation (5000 ×*g*, 4 °C, 5 min) followed by collection of clear supernatants. The combined filtrates of COLP extract and yogurt extracts were subjected to freeze drying and preservation at −80 °C for further use.

#### 2.4.2 Total phenolic content (TPC) determination

TPC of COLP and yogurt extracts was evaluated by the colorimetric method using Folin Ciocalteau (FC) reagent (Cho et al., 2017). A mixture of 0.2 mL extracts and 0.2 mL of 1 M FC was kept for 5 min at room temperature; subsequently, 0.6 mL of sodium carbonate was added, mixing well, and 30 min incubation of the reaction mixture at room temperature. Then, 2 mL ddH_2_O was added to the mixture and the absorbance was taken using a spectrophotometer (Lambda EZ 150, PerkinElmer, USA) at 725 nm and the findings were presented as milligram gallic acid equivalents (GAE) per gram of sample weight.

#### 2.4.3 DPPH antiradical activity measurement

DPPH antiradical activity assay (Jung et al., 2016) was utilized to examine the antioxidant activity of COLP and yogurt extracts. Concisely, the reaction mixture of 0.3 mL extract and 1.5 mL of DPPH solution (100 μM, in methanol) was incubated at room temperature in the dark for 15 min and then the absorbance (*A*) the blank and sample was measured spectrophotometrically at 518 nm and outcomes were stated as DPPH inhibition percentage as follow:

$$\text{DPPH inhbition}\;\left(\%\right)=\frac{A\;\left(\mathrm{blank}\right)-A\;\left(\mathrm{sample}\right)}{A\;\left(\mathrm{blank}\right)}\times 100$$

### 2.5 Acidity and pH measurement

The acidity yogurt sample was measured by titration against 0.1 N NaOH by the aid of phenolphthalein as an indicator as described in the official method (AOAC, 2005) and expressed as % lactic acid. The Thermo Orion pH meter model 420 (Thermo Scientifics, Waltham, MA, USA) was used for analysis of pH of yogurt samples. Triplicate yogurt samples were taken at day 1, 8, 16, and 24 of storage for analysis of pH and acidity.

### 2.6 Syneresis and water holding capacity (WHC) determination

Yogurt's syneresis and WHC were assessed after centrifugation of 10 *g* yogurt samples at 5000 ×g for 15 min at 4 °C followed by weighing the supernatant and precipitate separately for calculation of syneresis and WHC as follow:

$$\text{Syneresis}\;\left(\%\right)=\frac{\text{Supernatant weight}\;\left(g\right)}{\text{Yogurt sample weight}\;\left(g\right)}\times 100$$

$$\mathrm{WHC}\;\left(\%\right)=\frac{\text{Precipitate weight}\;\left(g\right)}{\text{Yogurt sample weight}\;\left(g\right)}\times 100$$

### 2.7 Texture profile analysis (TPA) and rheology measurement

The method designated by Saleh et al. (2020) was utilized for examination of yogurt texture using a TA/XT2 texture analyzer (Brookfield Engineering Laboratories, Inc., Middleboro, USA). A 5 mm diameter flat cylindrical probe was used to compress 10 g yogurt gel sample (at 4 °C) to 10 mm penetration depth at a 0.5 mm/s cross head speed, and at 25 °C (room temperature) and each yogurt sample was subjected to triplicate analyses. The rheology was examined using a HR-1 rheometer (TA Instruments, New Castle, PA, USA) as stated by Saleh et al. (2020). Concisely, 4 g yogurt was positioned on the plate (40 mm × 50 μm) and subjected to calibration at 25 °C for 2 min followed by collecting the data of dynamic shear at frequency swap of 0.1–10 rad/s and 1.0 Pa constant stress at 25 °C. Triplicate analyses were done and the results of storage moduli (G′) and loss moduli (G′′) that within the linear viscoelastic range was analyzed (software version 5.7.0, TA Instruments, New Castle, PA, USA).

### 2.8 Surface color measurement

A CR-300 Minolta colorimeter (Minolta Camera Co., Osaka, Japan) was used for analysis of CIE surface color traits (L*, a*, and b*) of yogurt sample after being calibrated by using a standard white plate. Triplicate analyses of each sample were taken and results were averaged.

### 2.9 Sensory evaluation

Thirty-five semi trained members (25 males and 10 females, age 22–36 years) were recruited from the students and staff of the College of Food and Agricultural Sciences, King Saud University to assess the sensory characteristics of yogurt. The members were chosen based on their interest and experience of sensory evaluation, regular consumption of yogurt (at least 3 times per week), non-smoking habits, and good health conditions. Prior to the analysis, the members received three training session about the samples and sensory parameters they supposed to score. The panel members were agreed voluntarily to involve in the study, fully informed about the study's procedure and purpose, gave an informed consent, and allowed to withdraw from the study at any time. No ethical approval was obtained and it was not required for this study. To protect the rights and privacy of all participants, appropriate protocols were implemented during the execution of this study. The evaluation was conducted in separate sensory evaluation booths and coded samples were arbitrarily served to the panel members and were requested use a 9-points hedonic scale (1 dislike extremely and 9 like extremely) for scoring the flavor, taste, texture, color, and overall acceptance of yogurt samples. The examinations were done in triplicates and the mean scores of the sessions and panelists of each sample were calculated and analyzed.

### 2.10 Statistical analysis

A completely randomized design was applied, and the data of three samples and evaluations were statistically analyzed using one-way ANOVA (SPSS software version 22, SPSS Inc., Chicago, IL, USA). Means comparison was done using Duncan's multiple range tests (DMRT) and *P* < 0.05 specify significance.

## 3 Result and discussion

### 3.1 Physicochemical properties of COLP

Cabbage outer leaves are an underutilized vegetable waste that generated in large volume on farms and markets creating significant impacts on the environment and economic losses. With the aim of adding value to this waste, this study evaluated the chemical composition of cabbage outer leaf powder (COLP) and its utilization in yogurt formulation. The results of chemical composition, bioactive properties, and color attributes of COLP are shown in Table 1. COLP is rich in vitamin C (156.62 mg/100 g), TPC (217 mg GAE/100 g), K (27,990 mg/ kg), Ca (6526.8 mg/kg), Na (8040 mg/ kg), Mg (2012.4 mg/ kg), Fe (124.92 mg/kg), TDF (64.41 g/100 g), IDF (52.91 g/100 g), and protein (17.30 g/100 g). It also exhibited high DPPH radical scavenging activity (82.81% inhibition). These findings indicate that COLP contain substantial amounts of nutrients and bioactive compounds and could be potentially used for development of functional foods. Previous reports showed that cabbage outer leaves are rich in nutrients with high potential food and pharmaceutical applications that is largely discarded as a waste in vegetable processing plant (Tanongkankit et al., 2010; Tawatsinlapasorn et al., 2017). According to Tanongkankit et al. (2010) cabbage outer leaves contain 18.43 g/100 g protein, 1.02 g/100 g lipids, 9.02 g/100 g ash, 40.89 g/100 g TDF, 33.54 g/100 g IDF, and 7.35 g/100 g SDF, which are partly consistent with our findings. Tanongkankit et al. (2010) reported that fresh and treated cabbage outer leaves contain 496.92–739.24 mg/100 g TPC and 76.52–82.94% DPPH radical scavenging activity, whereas Tawatsinlapasorn et al. (2017) stated that fresh and treated cabbage outer leaves contain 62.75–1800.2 mg/100 g TPC demonstrating a wide range of total phenolic content in cabbage outer leaves. Brito et al. (2021) observed that methanolic extract of free phenolic compounds of cabbage stalk flour has higher TPC and DPPH antiradical activity than pound phenolic extract and attributed that to the enzymatic and thermal hydrolysis processes that allowed the release of pound phenolic thus increasing their bioaccessibility and bioavailability. Zhao et al. (2024) reported that K is the most abundant microelement in cabbage leaves tailed by Ca and Na, whereas Fe is the most abundant microelement and the concentrations of these elements is greater in the external leaves than the internal leaves. In addition, they also observed that phenolic compounds and antioxidant activity were higher in the external leaves compared to the internal leaves and attributed that to the sunlight enhancement of the levels of phenolic compound synthesis enzymes namely coumarin-CoA and acetyl-CoA thereby producing more bioactive compounds in outer leaves (Zhao et al., 2024).

**Table 1:** Chemical composition of cabbage outer leaf powder (COLP).

Parameter	Mean ± SD
Moisture (g/100 g)	8.10 ± 2.11
Ash (g/100 g)	6.30 ± 0.52
Fat (g/100 g)	2.21 ± 0.10
Protein (g/100 g)	17.30 ± 1.12
Total dietary fiber (TDF, g/100 g)	64.41 ± 1.11
Insoluble dietary fiber (IDF, g/100 g)	52.91 ± 0.41
Soluble dietary fiber (SDF, g/100 g)	11.5 ± 0.91
Bioactive properties	
Vitamin C (mg/100 g)	156.62 ± 6.91
TPC (mg GAE/100 g)	217.06 ± 2.31
DPPH (%)	82.81 ± 1.33
Color	
L*	77.29 ± 0.31
a*	−1.88 ± 0.05
b*	15.38 ± 0.45
Minerals (mg/kg)	
K	27,990 ± 182.0
Ca	6526.8 ± 123.0
Na	8040 ± 164.0
Mg	2012.4 ± 102.0
Fe	124.92 ± 3.81
Mn	24.48 ± 3.41
Zn	23.04 ± 2.51
Cu	8.04 ± 1.36

Values are mean of three determinations ± standard deviation (n = 3).

### 3.2 Yogurt's chemical composition

Fortification of yogurt with different concentrations (0.5–2.5%) of COLP greatly (*p* < 0.05) enhanced yogurt's chemical composition as shown in Table 2. Increasing the concentration of COLP in yogurt formulation concomitantly (p < 0.05) augmented the ash, total solids, vitamin C, TDF, IDF, SDF, K, Ca, Na, Mg, Fe, and Mn of fortified yogurt reaching the maximum values of these attributes in yogurt fortified with 2.5% COLP. Compared to control yogurt, all COLP-fortified yogurt contains high amounts of dietary fiber, minerals, protein, and vitamin C demonstrating high nutritional and health values of COLP-fortified yogurt. This could be ascribed to high quantities of these nutrients in COLP as shown in Table 1. Similar observations on greater quantities of basic nutritional compounds in yogurts fortified with plant ingredients than control yogurts were reported by many investigators. Król et al. (2025) reported that the incorporation of freeze-dried cornflower petals into yogurt formulation significantly increased the total protein, crude fiber, vitamin C, Ca, K, and Mg of fortified yogurt related to control yogurt, and they attributed that to the richness of cornflower petals with these nutrients. Hafeez et al. (2025) reported that incorporation of carrot powder into goat yogurt increased the total solids, ash, fiber, vitamin C, Ca, K, Na, P, Zn, and Fe of the fortified yogurt compared to control ones. Salman et al. (2024) specified that fortification of probiotic yogurt with red beetroot juice enhanced the moisture, ash, Ca, K, and Na and reduced protein and fat content of the fortified yogurt compared to control ones. Bratkič et al. (2025)  reported similar tendencies of moisture, protein, ash, and minerals (K, Ca, Na, Mg, Mn, Zn, Fe, and Cu) in yogurt fortified with *Carpobrotus edulis* (L.) N. E. Br. fruit peel extracts compared to control yogurt. The increase in nutritional composition of yogurt fortified in the above studies is likely due to richness of added plant materials with these nutrients, the interaction of polyphenols in plant additives with milk macromolecules leading to formation of stable complexes and thereby increasing the total solids, and liberation of bound nutrients through metabolic activity of starter culture and thereby increasing the mineral contents of fortified yogurts (Salman et al., 2024; Zaki et al., 2024; Bratkič et al., 2025; Hafeez et al., 2025; Król et al., 2025).

**Table 2:** Chemical composition of yogurt fortified with cabbage outer leaf powder (COLP).

Parameter	Control	0.5% COLP	1.5% COLP	2.5% COLP
Total solids (%)	13.50 ± 0.5^c^	13.92 ± 0.5^c^	14.77 ± 0.5^b^	15.58 ± 0.5^a^
Ash (%)	0.72 ± 0.01^d^	0.77 ± 0.01^c^	0.86 ± 0.01^ab^	0.97 ± 0.02 ^A^
Fat (%)	3.52 ± 0.5^a^	3.53 ± 0.2^a^	3.53 ± 0.6^a^	3.54 ± 0.5^a^
Protein (%)	3.55 ± 0.4^b^	3.61 ± 0.7^a^	3.66 ± 0.3^a^	3.68 ± 0.5^a^
Vitamin C (mg/100 g)	3.27 ± 1.21^d^	9.81 ± 2.31^c^	13.08 ± 2.43^b^	16.35 ± 1.66^a^
TDF (%)	0.00 ± 0.00	0.32 ± 0.02^c^	0.98 ± 0.00^b^	1.63 ± 0.03 ^A^
IDF (%)	0.00 ± 0.00	0.26 ± 0.01^c^	0.78 ± 0.03^b^	1.36 ± 0.02 ^A^
SDF (%)	0.00 ± 0.00	0.06 ± 0.02^c^	0.19 ± 0.01^b^	**0.31 ± 0.02** ^**A**^

Minerals (mg/kg)				
K	16,560 ± 128^d^	21,420 ± 142.12^c^	21,990 ± 136^b^	22,540 ± 158.12 ^A^
Ca	6849.6 ± 122^d^	8379.6 ± 125^c^	10,177.2 ± 312^b^	10,417.2 ± 233^a^
Na	5370 ± 152.1^d^	6180 ± 124^c^	6360 ± 112^b^	6540 ± 196^a^
Mg	864.36 ± 23.21^c^	949.2 ± 52.31^b^	1243.2 ± 112^a^	1294.8 ± 142^a^
Fe	88.68 ± 2.31^d^	100.44 ± 3.21^c^	155.4 ± 4.23^b^	201 ± 3.62^a^
Zn	32.04 ± 2.31^d^	35.4 ± 1.96^c^	41.52 ± 1.78^a^	40.56 ± 2.11^b^
Mn	5.76 ± 1.21^d^	8.52 ± 1.53^c^	11.64 ± 2.11^b^	17.4 ± 1.65^a^

Values are mean of three determinations ± standard deviation (n = 3) Values followed by different letters within each row are significantly different (p < 0.05).

### 3.3 Yogurt's bioactive properties

The outcomes of TPC and DPPH antiradical activity of yogurt fortified with different concentrations (0%, 0.5%, 1.5%, and 2.5%) of COLP and stored at 4 °C for 1, 8, 16, and 24 days are presented in Table 3. COLP yogurt displayed greater (*p* < 0.05) TPC and DPPH radical inhibition than control yogurt. Increasing COLP level in yogurt formulation concurrently (p < 0.05) augmented the TPC and DPPH antiradical activity and the maximum levels were seen in yogurt fortified with 2.5% COLP, which is likely due to high bioactive properties of COLP in addition to the release of bound bioactive compounds from COLP during fermentation processes. Similarly, a concomitant upsurge in the TPC and DPPH antiradical activity of set-type yogurts fortified with white radish powder (Mohamed Ahmed et al., 2024), semi-defatted walnut and pumpkin flour (Trajkovska et al., 2025), olive leaf extract (Tarchi et al., 2025), green pepper extract (Kovsari et al., 2024) and carrot powder (Hafeez et al., 2025) was reported and attributed to higher TPC and DPPH antiradical activity in these plant ingredients. Throughout storage, the TPC and DPPH antiradical activity of all yogurts were concomitantly (p < 0.05) reduced as storage time lengthened attaining the least amounts at the end of storage (24 days). It is worth noting that the DPPH antiradical activity and TPC of yogurt fortified with 1.5% and 2.5% COLP at the end of storage were significantly higher in comparison with fresh control yogurt at the beginning of storage, suggesting enhanced bioactive properties of COLP-containing yogurt throughout the storage period. A simultaneous reduction in TPC and DPPH radical-scavenging activity was noted during the storage of yogurt enriched with argel leaf extract (Mohamed Ahmed et al., 2020), phenolic-rich ingredients of Isabel grape (Silva et al., 2022), white radish powder (Mohamed Ahmed et al., 2024), and carrot powder (Hafeez et al., 2025). However, a rise in the TPC and DPPH antioxidant activity was also reported during storage of yogurt fortified with alfalfa sprout flour (Ghaderi-Ghahfarokhi et al., 2024), orange peel extract (Zaki et al., 2024), pumpkin and walnut semi-defatted flour (Trajkovska et al., 2025), and olive leaf extract (Tarchi et al., 2025), which was attributed to the discharge of bound bioactive compounds from yogurt network structure by hydrolysis of cell wall component enzymatically and non-enzymatically thereby increase the extractability and availability of bioactive compounds and consequently increase antioxidant activity during storage. The decrease in TPC and DPPH antiradical activity of yogurt throughout storage is possibly owed to the formation of complexes between bioactive compounds and yogurt matrix and thereby diminish their solubility and extractability, metabolism of bioactive compounds by LAB, and involvement of bioactive compounds in antioxidative mechanisms to counteract the protein and lipid oxidation (Mohamed Ahmed et al., 2024; Silva et al., 2022).

**Table 3:** Total phenolic contents (TPC, mg GAE/ 100 g) and antioxidant (DPPH inhibition, %) activity of yogurt sample fortified with cabbage outer leaf powder (COLP) during cold storage at 4 °C.

Parameters	Storage (days)	Yogurt fortified with COLP			
		Control	0.5% COLP	1.5% COLP	2.5% COLP
TPC (mg GAE/ 100 g)					
	1	21.38 ± 0.61 ^A, d^	32.46 ± 0.12 ^A, c^	63.14 ± 0.14 ^A, b^	74.33 ± 2.13 ^A, a^
	8	20.41 ± 0.48^B, d^	30.77 ± 0.31^B, c^	58.18 ± 0.28^B, b^	73.01 ± 1.61^B, a^
	16	17.34 ± 0.28^C, d^	27.94 ± 0.92^C, c^	50.10 ± 0.28^C, b^	67.82 ± 2.51^C, a^
	24	13.80 ± 0.14^D, d^	19.21 ± 0.81^D, c^	46.79 ± 1.28^D, b^	58.57 ± 1.31^D, a^

DPPH (% inhibition)					
	1	22.60 ± 1.20 ^A, d^	33.52 ± 2.02 ^A, c^	46.32 ± 2.12 ^A, b^	62.41 ± 1.22 ^A, a^
	8	20.00 ± 1.01^B, d^	30.10 ± 1.06^B, c^	45.41 ± 1.62^B, b^	62.00 ± 2.12 ^A, a^
	16	15.50 ± 1.20^C, d^	22.68 ± 1.01^C, c^	40.12 ± 1.14^C, b^	55.44 ± 1.32^B, a^
	24	8.16 ± 1.15^D, d^	14.43 ± 2.01^D, c^	34.13 ± 1.51^D, b^	42.39 ± 1.61^C, a^

Values are presented as mean ± standard deviation. A − D Means followed by different uppercase letters in the same column are significantly different (p < 0.05). a-c Means followed by different lowercase letters in the same row for the same parameter are significantly different (*p* < 0.05).

### 3.4 Yogurt's pH, acidity, WHC, and syneresis

**Fig. 1:**
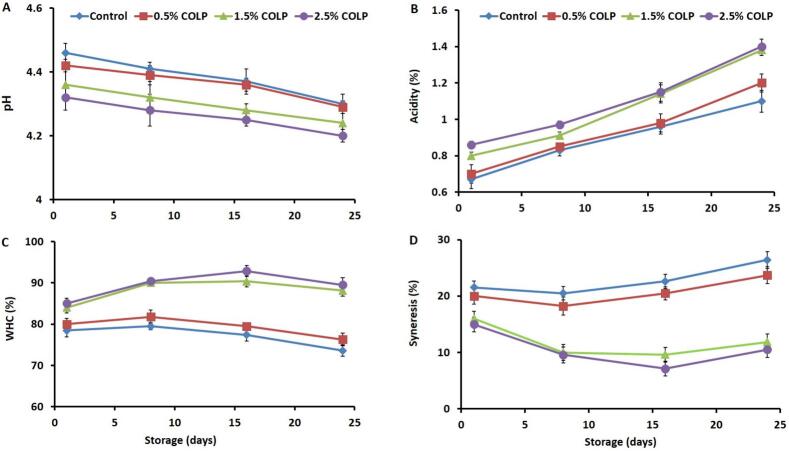
pH (A), acidity (B), water-holding capacity (C), and syneresis (D) of yogurt fortified with 0% COLP (Control), 0.5% COLP, 1.5% COLP, and 2.5% COLP during 24 days of storage at 4 °C.

The results of pH, acidity, WHC, and syneresis of yogurt fortified with different levels of COLP are depicted in Fig. 1a–d. The pH of control yogurt and 0.5% COLP yogurt samples was higher than that of 1.5% and 2.5% COLP-containing yogurts throughout the storage (Fig. 1a). Throughout storage, the pH of all yogurts showed concurrent reduction attaining the smallest values at the end of storage (24 days). The acidity of control yogurt and 0.5% COLP yogurt samples was greatly (*p* < 0.05) lesser compared to that of 1.5%and 2.5% COLP-containing yogurts throughout the storage (Fig. 1b). Yogurt's acidity was simultaneously (p < 0.05) augmented as the storage time elongated reaching the highest levels at the end of storage time (24 days). These findings demonstrate that incorporation of 1.5% and 2.5% COLP into yogurt preparation expressively reduce the pH and increase the acidity throughout the storage (Fig. 1a and b). The drop of pH and upsurge of acidity of 1.5% and 2.5% COLP yogurts is likely due to the fermentation of fermentable components in COLP by the starter culture of LAB, which might result in the production of organic acids and thus increase the pH and reduce the acidity of fortified yogurts (Mohamed Ahmed et al., 2024). In addition, phenolic and flavonoids compounds in COLP could enhance the metabolic activity of LAB and thereby increasing the acidity and reducing pH of fortified yogurts (Zhang et al., 2019). Similar changing trends in the acidity and pH were noticed following the incorporation of Isabel grape ingredient (Silva et al., 2022), alfalfa sprout flour (Ghaderi-Ghahfarokhi et al., 2024), white radish powder (Mohamed Ahmed et al., 2024), orange peel extract (Zaki et al., 2024), and carrot powder (Hafeez et al., 2025) into yogurt formulations and attributed that to enhancing the metabolic activity of LAB by bioactive and fermentable components of added plant materials and consequently rising the acidity and dropping the pH of fortified yogurts compared to plain ones. Acidity contributes to the textural properties, sensory attributes, and health benefits of yogurts and is greatly influenced by factors such as raw materials, type of LAB culture, processing and storage conditions, and yogurt formulations (Karaca et al., 2025). The pH and acidity ranges of plain and COLP-fortified yogurts in this study were within the standard pH (≤ 4.6) and acidity (0.6–1.5% lactic acid) limits of fermented dairy products (Alimentarius, 2015; Ferreira & Santos, 2023) signifying the suitability of developed yogurts for human consumption. Incorporation of COLP into yogurt formulations at the concentrations of this study not only add value to this essential waste, but also can enhance the acidity and the sensory quality of the develop yogurt.

WHC of yogurt increased following the increasing concentration of the COLP in yogurt formulation with the highest levels of WHC being seen in 2.5% COLP yogurt (Fig. 1c). No differences in the WHC between control and 0.5% COLP yogurts and between 1.5% and 2.5% COLP yogurts. However, the latter differed significantly (*p* < 0.05) from the former. During storage, WHC of control and 0.5% COLP yogurt gradually reduced to the lowest levels at the end of storage, whereas, that of 1.5% and 2.5% COLP augmented to the highest levels at day 16 of storage followed by a slight reduction at day 24. The syneresis of control and 0.5% COLP yogurts were greater (p < 0.05) than that of yogurt fortified with 1.5% and 2.5% CLOP throughout the whole storage duration (Fig. 1d). The syneresis was reduced during the first week of storage and slightly increased for control and 0.5% COLP yogurts, remained constant for 1.5% COLP yogurts, and further reduced at day 16 and then slightly increased for 2.5% COLP yogurts. The increase in WHC and reduction of syneresis of yogurts fortified with 1.5% and 2.5% COLP is probably owing to the development of stronger gel network between COLP polyphenols and milk proteins, modification of casein network, increasing total solids, and absorption of water by porous fibrillar structures of COLP (Dong et al., 2022; Kwon et al., 2019; Mohamed Ahmed et al., 2024). Similarly, improved WHC and reduced syneresis were observed in yogurts fortified with white mulberry powder (Sheikh et al., 2023), alfalfa sprout flour (Ghaderi-Ghahfarokhi et al., 2024), white radish powder (Mohamed Ahmed et al., 2024), and carrot powder (Hafeez et al., 2025). In addition, higher WHC was reported in goat yogurt fortified with fiber, protein, starch, and germ powder samples of pea compared to control ones (Qin et al., 2025). The changing trends of WHC and syneresis of yogurt with and without COLP throughout storage could be accredited to reorganization and reconstruction of the casein network and intensification of yogurt's acidity during storage (Yousefi et al., 2025). The results of this study demonstrate that incorporation of COLP into yogurt formulations improve the WHC and syneresis and would therefore enhance the overall quality and stability of fortified yogurt.

### 3.5 Yogurt's texture profile

The textural properties of yogurt fortified with varied amounts of COLP is presented in Table 4. Incorporation of COLP into yogurt formulation increased the hardness, adhesiveness, springiness, and cohesiveness properties of the fortified yogurt at different magnitudes compared to control yogurt. The greatest (*p* < 0.05) values of hardness, cohesiveness, springiness, and adhesiveness were noticed in yogurt fortified with 1.5% and 2.5% COLP as compared to that of control and 0.5% COLP yogurt. During storage, the levels of hardness, cohesiveness, springiness, and adhesiveness were augmented as the storing time elongated achieving the highest values at the end of storage with few exceptions. The interaction of COLP fiber and milk protein that lead to stronger milk gel structure, the increase in total solid contents, the increase in WHC and acidity, and metabolic action of LAB might be among the reasons for enhanced textural properties of COLP containing yogurts. Comparable to our results, Zhang et al. (2024) informed that incorporation of kale fiber into goat yogurt increased the cohesiveness, hardness, adhesiveness, gumminess, and chewiness and attributed that to the rise in total solids and the interaction of fiber and milk protein. In addition, Qin et al. (2023) stated that grape fruit high-quality dietary fiber enhanced the hardness of yogurt due to the stabilization of casein-pectin structure by electrostatic interaction leading to the formation of stable gel. Moreover, Qin et al. (2025) reported that addition of pea ingredients (fiber powder, starch powder, germ powder, and protein powder) into goat yogurt increased the hardness, cohesiveness, and adhesiveness compared to control and attributed that to increase in WHC and formation a robust amylose network causing more stable gel structure. Furthermore, Wang et al. (2025) reported that incorporation of hawthorn powder into yogurt improved the hardness and cohesiveness and attributed that to presence of polyphenols and pectin in hawthorn powder that could promote protein cross-linking. Overall, addition of COLP can boost the textural quality of yogurt and consequently the physical and sensory quality attributes of yogurt.

**Table 4:** Texture profile analysis (TPA) of yogurt formulated with the various concentrations of cabbage outer leaf powder (COLP) during storage at 4 °C.

Texture Profile Analysis (TPA)	Storage (days)	Yogurt fortified with COLP			
		Control	0.5% COLP	1.5% COLP	2.5% COLP
Hardness (g)					
	1	14.0 ± 0.01^D, d^	15.0 ± 0.02 ^D, c^	16.0 ± 0.05 ^D, a^	17.0 ± 0.09 ^D, a^
	8	16.0 ± 0.11^C, d^	18.0 ± 0.20^C, c^	21.0 ± 0.14^C, b^	23.0 ± 0.10^C, a^
	16	18.0 ± 0.12 ^B, d^	20.0 ± 0.01^B, c^	24.0 ± 0.02 ^B, b^	26.0 ± 0.20 ^B, a^
	24	22.0 ± 0.13 ^A, d^	25.0 ± 0.03 ^A, c^	29.0 ± 0.04 ^A, b^	32.0 ± 0.10 ^A, a^

Cohesiveness					
	1	0.33 ± 0.02^C, b^	0.34 ± 0.01^C, b^	0.36 ± 0.01^C, a^	0.37 ± 0.01 ^D, a^
	8	0.36 ± 0.01 ^B, b^	0.37 ± 0.01 ^B, b^	0.38 ± 0.01^C, ab^	0.40 ± 0.01^C, a^
	16	0.37 ± 0.01^B, c^	0.38 ± 0.01 ^B, c^	0.40 ± 0.00 ^B, b^	0.45 ± 0.03 ^B, a^
	24	0.40 ± 0.01 ^A, d^	0.44 ± 0.02 ^A, c^	0.50 ± 0.02 ^A, b^	0.56 ± 0.02 ^A, a^

Springiness (mm)					
	1	7.40 ± 0.01^C, c^	9.20 ± 0.01^B, b^	9.50 ± 0.02 ^D, a^	9.50 ± 0.10^C, a^
	8	8.20 ± 0.01^B, d^	9.90 ± 0.08 ^AB, b^	10.80 ± 0.04^C, a^	9.70 ± 0.05 ^B, c^
	16	9.60 ± 0.02 ^A, c^	10.10 ± 0.06 ^A, b^	11.10 ± 0.01^B, a^	10.30 ± 0.20 ^A, b^
	24	9.50 ± 0.02 ^A, d^	10.20 ± 0.03 ^A, c^	13.00 ± 0.05 ^A, a^	10.60 ± 0.20 ^A, b^

Adhesiveness					
	1	0.10 ± 0.01^B, d^	0.20 ± 0.02 ^B, c^	0.40 ± 0.01^C, a^	0.30 ± 0.02 ^D, b^
	8	0.20 ± 0.02 ^B, c^	0.20 ± 0.01 ^B, c^	0.40 ± 0.06^C, a^	0.30 ± 0.01^C, b^
	16	0.30 ± 0.01 ^A, c^	0.30 ± 0.01 ^A, c^	0.50 ± 0.02 ^B, a^	0.40 ± 0.01^B, b^
	24	0.10 ± 0.01^C, d^	0.20 ± 0.04 ^B, c^	0.80 ± 0.03 ^A, a^	0.70 ± 0.01 ^A, b^

Values are presented as mean ± standard deviation. A − D Means followed by different uppercase letters in the same column are significantly different (p < 0.05). a-c Means followed by different lowercase letters in the same row for the same parameter are significantly different (p < 0.05).

### 3.6 Yogurt's rheological characteristics

**Fig. 2:**
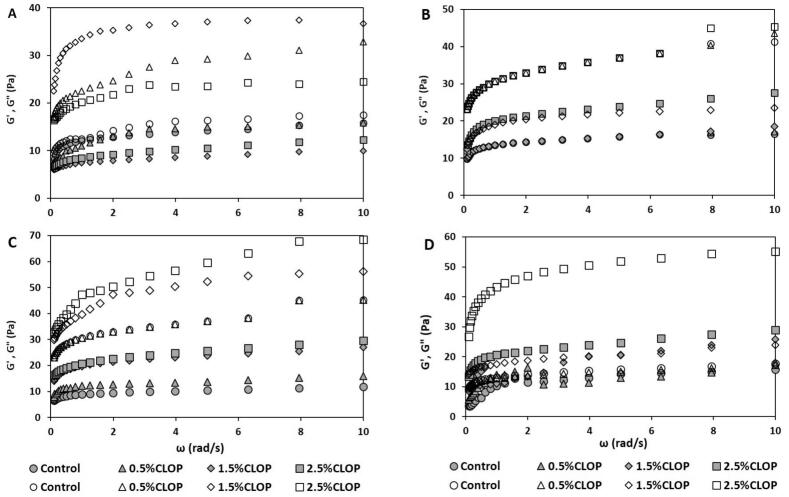
Rheological parameters G' (open), G" (solid) of yogurt fortified with 0% COLP (Control, circle symbol), 0.5% COLP (triangle symbol), 1.5% COLP (diamond symbol), and 2.5% COLP (squire symbol) at day 1 (A), day 8 (B), day 16 (C), and day 24 (D) of storage at 4 °C.

The rheological attributes of yogurt, particularly the storage modulus (G', elasticity) and loss modulus (G", viscosity), offer important insights into its internal structure (Mohamed Ahmed et al., 2021). Fig. 2a–d displays the rheological properties of yogurt enriched with 0%, 0.5%, 1.5%, and 2.5% COLP. Commonly, weak viscoelastic gels (G' > G”) behavior were noticed in all yogurts suggesting the solid like structure of the formed yogurt and therefore good storage stability. Addition of COLP to yogurt formulation expressively (p < 0.05) affected G' and G" with the highest effects observed in 1.5% and 2.5% COLP yogurts (Fig. 2a–d). The storage durations affected G' and G" at different magnitudes. At the beginning of storage, there were noteworthy variation in the levels of G' and G" among yogurt types (Fig. 2a). At day 8 and day 24 of storage, there are slight changes in the G' and G" with the highest values being observed in 2.5% COLP yogurt (Fig. 2b and d). Significant differences in the G' and G" between yogurt samples were observed with higher levels in 1.5% and 2.5% COLP yogurts (Fig. 2c). Generally, the firmness of yogurt augmented with upsurge in COLP levels and storage duration. The highest G' in 1.5% and 2.5% COLP yogurts throughout the storage proved stronger gels with improved elastic behavior compared control and 0.5% COLP yogurts and thus suggested the effectiveness of COLP at 1.5 and 2.5% in forming yogurts with stable structure. The fortified yogurts have higher G' than G" throughout the storage suggesting their solid-like structure, better resistance to deformation and phase separation, lower syneresis, and smooth and cohesive mouthfeel and consequently better storage stability and consumer acceptability (Greis et al., 2022; Le Ba et al., 2025). The improved gel elasticity at 1.5% and 2.5% COLP is likely resulted from interactions of COLP polyphenols and starch with milk casein, fiber self-entanglement, and enhanced organic acid production by LAB, leading to stronger three-dimensional network structures (Mohamed Ahmed et al., 2024). In addition, COLP soluble fiber may form electrostatic and hydrogen bonds with casein in fortified yogurt leading to the creation of densely packed gel network, solid-gel-like structure, and firm and stable yogurts (Khubber et al., 2021). Moreover, COLP insoluble fiber may interact with yogurt casein through cross-linking, filling, and bridging effects and forms COLP-casein clusters and more stable and dense yogurts gel network structure (Wang et al., 2025). In agreement with our findings, Qin et al. (2023) informed that incorporation of grapefruit dietary fiber into goat yogurt enhanced the G' and G" and improving the gel network structure of fortified yogurt compared to control. Additionally, Qin et al. (2025) specified that goat yogurt fortified with 0.5% pea fiber, pea starch powder, pea germ powder, and pea protein powder had the highest modulus (G' and G") levels compared to control yogurts. Moreover, Yousefi et al. (2025) reported addition of *Plantago major* L. seed mucilage to low-fat yogurt at 1%, 3% and 5% concomitantly increased G' and G" levels compared to control yogurt and accredited that to the interaction of added mucilage with casein micelles leading to increased strength and elasticity of yogurt gel. Furthermore, Mohamed Ahmed et al. (2024) reported that incorporation of 2% white radish powder into yogurt enhanced the G´ values compared to control and they attributed that to the interaction of polyphenols of white radish powder with milk casein forming a solid gel matrix. They also observed that incorporation of 4% white radish powder into yogurt reduced G´ levels compared to control that is likely owing to the interaction insoluble components in white radish powder with milk protein and consequently hinder gel network formation. From an industrial perspective, the enhanced elastic behavior (higher G′) observed at 1.5% COLP suggests improved gel robustness during pumping, filling, and cold storage, which is desirable for large-scale yogurt production. However, higher fiber levels (*e.g.*, 2.5%) may require optimization of processing parameters such as homogenization pressure and fermentation time to ensure uniform dispersion and avoid excessive firmness. Therefore, incorporating COLP at approximately 1.5% appears to offer a balance between improved rheological stability and processability under industrial yogurt manufacturing conditions. Overall, COLP could enhance the rheological quality attributes of yogurt throughout cold storage.

### 3.7 Color properties yogurts

**Fig. 3:**
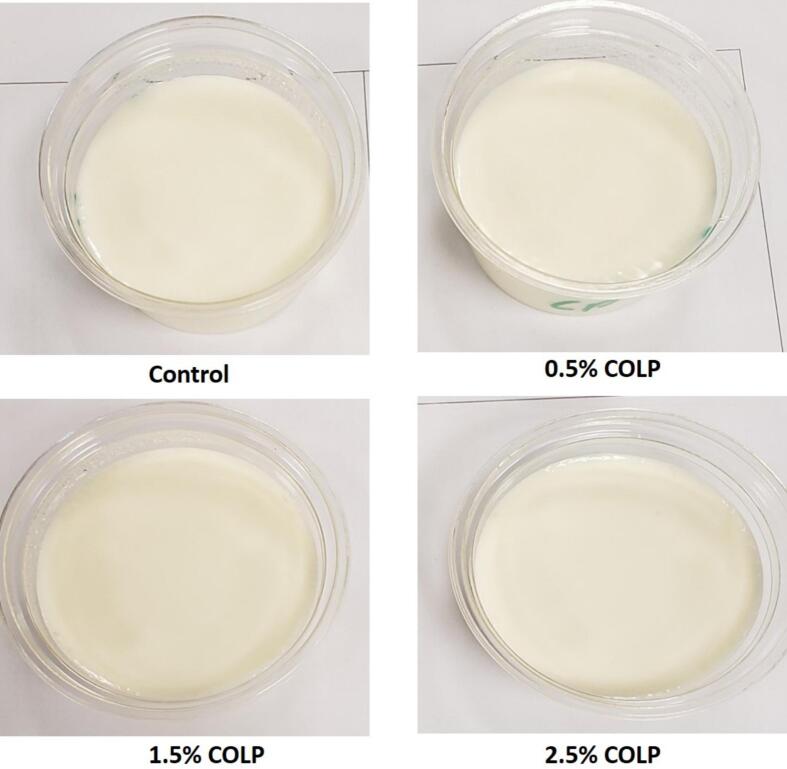
Photograph of yogurt fortified with 0% (control) COLP, 0.5% COLP, 1.5% COLP, and 2.5%COLP.

Color is one of the most important characteristics that critically affects the consumer acceptance of yogurt (Spence, 2023). The surface color properties of yogurt fortified with diverse concentrations of COLP is shown in Table 5 and Fig. 3. Incorporation of COLP into yogurt formulation affected the surface color of the product in different manners. It declined the lightness (L*) and augmented the yellowness (b*) and redness (a*) of COLP-fortified yogurts compared to control ones. The reduction of lightness of COLP-containing yogurts may be attributed to high WHC of fortified yogurts that capture free water and thereby reduce light reflection from yogurt surface due to less free water on the surface (Jrad et al., 2019). The intensification in redness and yellowness of COLP-yogurts is likely due to the existence of some natural colorings such as carotenoids, chlorophylls, and riboflavin in COLP (Mohamed Ahmed et al., 2024). Throughout storage the L* and b* values reduced concomitantly as the storage time advanced, while, a* values showed an increase as storage time elongated. The changes in lightness, redness, and yellowness throughout storage might be attributed to the modification of gel network structure and changes acidity of yogurt by LAB leading to the release of natural dyes into the matrix or surface of yogurt as the storage progressed (Costa et al., 2015). Mohamed Ahmed et al. (2024) reported similar trends as the incorporation of white radish powder into yogurt decreased the lightness and augmented yellowness and redness of enriched yogurt compared to control. In addition, increased in a* and b* values and reduction of L* values were reported for yogurt fortified with carrot soluble dietary fiber (Dong et al., 2022), grape fruit dietary fiber (Qin et al., 2023), modified okara insoluble dietary fiber (Tian et al., 2024), olive leaf extract (Tarchi et al., 2025), semi-defatted walnut and pumpkin flour (Trajkovska et al., 2025), and hawthorn powder (Wang et al., 2025).

**Table 5:** Surface color characteristics of yogurt formulated with the various concentrations of cabbage outer leaf powder (COLP) during storage at 4 °C.

Color properties	Storage (days)	Yogurt fortified with COLP			
		Control	0.5% COLP	1.5% COLP	2.5% COLP
L*					
	1	78.51 ± 0.01 ^A, a^	75.50 ± 0.02 ^A, c^	77.31 ± 0.05 ^A, b^	77.34 ± 0.03 ^A, b^
	8	78.50 ± 0.05 ^A, a^	73.50 ± 0.01^B, c^	77.31 ± 0.01 ^A, b^	77.33 ± 0.10 ^A, b^
	16	66.48 ± 0.01^B, d^	70.46 ± 0.01^C, b^	75.83 ± 0.01^B, a^	67.28 ± 0.09 ^B, c^
	24	62.45 ± 0.01^C, c^	65.43 ± 0.05^D, b^	73.61 ± 0.01^C, a^	60.89 ± 0.09^C, d^

a*					
	1	−2.12 ± 0.05^B, c^	−1.95 ± 0.04^B, b^	−2.00 ± 0.06^B, c^	−1.78 ± 0.00^C, a^
	8	−2.00 ± 0.06^B, c^	−1.90 ± 0.01^B, b^	−2.08 ± 0.02^B, c^	−1.70 ± 0.01^B, a^
	16	−1.92 ± 0.03 ^A, c^	−1.82 ± 0.01 ^A, b^	−2.04 ± 0.01^B, d^	−1.68 ± 0.01^B, a^
	24	−1.86 ± 0.01 ^A, c^	−1.77 ± 0.03 ^A, b^	−1.89 ± 0.02 ^A, c^	−1.62 ± 0.01 ^A, a^

b*					
	1	5.10 ± 0.04 ^A, d^	6.41 ± 0.01 ^A, c^	7.57 ± 0.01 ^A, b^	9.31 ± 0.01 ^A, a^
	8	5.09 ± 0.02 ^A, d^	6.39 ± 0.01 ^A, c^	7.50 ± 0.01^B, b^	9.20 ± 0.01^B, a^
	16	5.00 ± 0.01^B, d^	6.36 ± 0.05 ^A, c^	7.47 ± 0.02^B, b^	8.92 ± 0.01^C, a^
	24	4.91 ± 0.01^C, d^	6.26 ± 0.01^B, c^	7.45 ± 0.00 ^B, b^	8.55 ± 0.02^D, a^

Values are presented as mean ± standard deviation. A − D Means followed by different uppercase letters in the same column are significantly different (p < 0.05). a-c Means followed by different lowercase letters in the same row for the same parameter are significantly different (p < 0.05).

### 3.8 Sensory attributes of yogurts

The sensory attributes of yogurt fortified with diverse levels of COLP are exposed in Table 6. Addition of COLP to yogurt impact the sensory qualities of the product at different magnitudes. Generally, incorporation of 2.5% COLP into yogurt formulation reduced the taste, flavor, color, and overall acceptability and increased the texture of fortified yogurt compared to control yogurt. No significant differences in all sensory features were evident among control yogurt and those fortified with 0.5% and 1.5% COLP suggesting that incorporation of 0.5% and 1.5% COLP into yogurt did not alter the sensory characteristics of fortified yogurts. All sensory attributes were concomitantly reduced as the storage time elevated. It is worth noting that all sensory attributes received scores higher than the cutoff score (5.0) with exceptions of control and 2.5% COLP yogurts stored for 24 days suggesting that incorporation of 0.5% or 1.5% COLP into yogurt could preserve the sensory qualities of fortified yogurt during cold storage. The scores of all sensory attributes were inside the satisfactory range (4–9) of sensory scores of yogurts (Tamime & Robinson, 1999).

**Table 6:** Sensory analysis of yogurt formulated with the various concentrations of cabbage outer leaf powder (COLP) during storage at 4 °C.

Sensory properties	Storage (days)	Yogurt fortified with COLP			
		Control	0.5% COLP	1.5% COLP	2.5% COLP
Color					
	1	8.30 ± 0.18 ^A, a^	8.20 ± 0.12 ^A, a^	7.91 ± 0.39 ^A, a^	6.13 ± 0.31 ^A, b^
	8	8.20 ± 0.48 ^A, a^	8.11 ± 0.18 ^A, a^	7.96 ± 0.17 ^A, a^	6.18 ± 0.10 ^A, b^
	16	6.82 ± 0.50^B, a^	6.55 ± 0.17^B, a^	7.00 ± 0.18^B, a^	5.73 ± 0.21^B, b^
	24	4.86 ± 0.18^C, c^	5.33 ± 0.20^C, b^	6.00 ± 0.12^C, a^	5.42 ± 0.11^B, b^

Flavor					
	1	7.50 ± 0.27 ^A, a^	7.17 ± 0.15 ^A, a^	7.20 ± 0.33 ^A, a^	6.11 ± 0.11 ^A, b^
	8	7.17 ± 0.20 ^A, a^	7.16 ± 0.18 ^A, a^	7.11 ± 0.10 ^A, a^	6.00 ± 0.10 ^A, b^
	16	6.17 ± 0.30^B, a^	6.55 ± 0.14^B, a^	6.57 ± 0.14^B, a^	5.13 ± 0.11^B, b^
	24	4.66 ± 0.11^C, b^	5.05 ± 0.12^C, a^	5.11 ± 0.18^C, a^	4.16 ± 0.21^C, c^

Taste					
	1	8.16 ± 0.18 ^A, a^	8.10 ± 0.20 ^A, a^	8.11 ± 0.15 ^A, a^	7.45 ± 0.23 ^A, b^
	8	8.00 ± 0.11 ^A, a^	7.56 ± 0.38 ^A, a^	7.70 ± 0.28 ^A, a^	6.12 ± 0.21^B, b^
	16	6.96 ± 0.12^B, a^	6.83 ± 0.10^B, a^	7.12 ± 0.24^B, a^	5.10 ± 0.11^C, b^
	24	4.11 ± 0.30^C, c^	5.13 ± 0.20^C, b^	6.62 ± 0.41^C, a^	4.50 ± 0.21^D, c^
					
Texture					
	1	7.26 ± 0.28 ^A, b^	8.00 ± 0.18 ^A, a^	8.20 ± 0.11 ^A, a^	8.19 ± 0.12 ^A, a^
	8	7.16 ± 0.19 ^A, b^	7.85 ± 0.18 ^A, a^	8.00 ± 0.14 ^A, a^	8.16 ± 0.20 ^A, a^
	16	6.59 ± 0.18^B, c^	7.21 ± 0.12^B, b^	7.76 ± 0.28^B, a^	8.00 ± 0.11 ^A, a^
	24	4.89 ± 0.10^C, c^	6.14 ± 0.22^C, b^	6.97 ± 0.10^C, a^	7.41 ± 0.31^B, a^

Overall acceptability					
	1	8.10 ± 0.10 ^A, a^	8.00 ± 0.18 ^A, a^	8.00 ± 0.18 ^A, a^	7.50 ± 0.11 ^A, b^
	8	7.13 ± 0.11^B, a^	7.00 ± 0.18^B, a^	7.00 ± 0.16^B, a^	6.00 ± 0.21^B, b^
	16	6.23 ± 0.10^C, a^	6.55 ± 0.12^C, a^	6.40 ± 0.10^C, a^	5.50 ± 0.21^C, b^
	24	5.22 ± 0.10^D, b^	5.67 ± 0.10^D, a^	5.36 ± 0.13^D, b^	4.50 ± 0.18^D, c^

Values are presented as mean ± standard deviation. A − D Means followed by different uppercase letters in the same column are significantly different (p < 0.05). a-c Means followed by different lowercase letters in the same row for the same parameter are significantly different (p < 0.05).

## 4 Conclusion

This study demonstrates that COLP is rich in bioactive compounds, minerals, and dietary fiber, possess antioxidant activity, and provide natural dietary fiber, bioactive properties, and storage stability to yogurt. Incorporation of COLP different concentrations (0%, 0.5%, 1.5%, and 2.5% COLP) into yogurt formulation enhanced the nutritional composition, bioactive properties, and physical quality attributes of fortified yogurt compared to control. These includes increasing mineral contents, vitamin C, dietary fiber, TPC, DPPH antiradical activity, WHC, hardness, cohesiveness, springiness, adhesiveness, and color attributes of COLP-containing yogurt compared to control. COLP formed stronger gels with improved elastic behavior of fortified yogurt during the entire storage. COLP also improved and preserved the physical, nutritional, and sensory quality characteristics of yogurt throughout cold storage for 24 days due to its antioxidant activity and preservative properties. Overall, incorporation of 1.5% COLP into yogurt formulation can enhance the physicochemical, nutritional, and sensorial quality attributes of yogurt. Thus, COLP is a natural additive for the production of functional yogurt that satisfies consumer demands and provides health benefits as well as adding values to this wasted vegetable byproduct. However, using one type of starter culture, conducting sensory analysis with semi-trained panelists, missing the analysis of total viable counts, yeasts and molds, and *in vivo* studies of COLP-fortified yogurt are limitations of this study. Future studies should address these aspects to prove the functional claims of COLP-fortified yogurt. In addition, optimization yogurt processing conditions could promote large scale production and commercialization of COLP-fortified yogurt.

## CRediT authorship contribution statement

**Suleiman A. Althawab:** Writing – review & editing, Writing – original draft, Visualization, Validation, Resources, Funding acquisition, Conceptualization. **Hesham Al-Quh:** Methodology, Investigation, Formal analysis, Data curation. **Abdulhakeem Alzahrani:** Writing – review & editing, Visualization, Validation. **Isam A. Mohamed Ahmed:** Writing – review & editing, Writing – original draft, Software, Resources, Investigation, Conceptualization.

## Funding

This research is funded by Waed Program (W25–12), King Saud University, Riyadh, Saudi Arabia.

## Declaration of competing interest

The authors declare that they have no known competing financial interests or personal relationships that could have appeared to influence the work reported in this paper.

## Data Availability

Data will be made available on request.
